# A Field Guide to Genomics Research

**DOI:** 10.1371/journal.pbio.1001744

**Published:** 2014-01-07

**Authors:** Andrea H. Bild, Jeffrey T. Chang, W. Evan Johnson, Stephen R. Piccolo

**Affiliations:** 1 Department of Pharmacology and Toxicology, University of Utah, Salt Lake City, Utah, United States of America; 2 Department of Oncological Sciences, University of Utah, Salt Lake City, Utah, United States of America; 3 Department of Integrative Biology and Pharmacology, Medical School, School of Biomedical Informatics, University of Texas Health Science Center at Houston, Houston, Texas, United States of America; 4 Division of Computational Biomedicine, Boston University School of Medicine, Boston, Massachusetts, United States of America; University of California Davis, United States of America

## Abstract

Portraying high-throughput genomics research as a wild frontier, Andrea Bild and colleagues use caricatures to highlight common pitfalls in genomic research and provide recommendations for navigating this terrain.

High-throughput technologies are enabling scientists to profile genomes, transcriptomes, proteomes, and metabolomes at an unprecedented scale [1]. All this “-omics” research (let's call it *genomics* for simplicity) is exciting—and game changing—but it's also fraught with dangers for the tenderfoot. Here, we've put together a brief “field guide” for those wishing to visit the genomics frontier, in which we use caricatures to illustrate various pitfalls that can beset those who inhabit this new territory. By documenting the behaviors of these common types, we hope to guide researchers in their quest to apply sound practices when designing genomics experiments and analyzing the resulting data. Many of the tendencies we have encountered are not specific to genomics research, but they are particularly acute in this field due to its interdisciplinary nature and the complexity of the data it produces.

Drawing upon our own experiences in various roles on genomics projects—and at the risk of generalizing—we note the prevalence of no fewer than six different character traits that lead to problems in experimental design (“the farmer”), data interpretation (“the gold miner” and “the cowboy”), collaboration (“the hermit” and “the master and servant”), and civic virtue (“the jailer”). This list is not comprehensive, but we hope it will guide new adventurers in the approaches and attitudes needed to stake their claim in this novel terrain.

## The Farmer

*“Let's harvest a bunch of data and design fancy tools, and then we'll figure out what to do with them.”*

**Figure 1:**
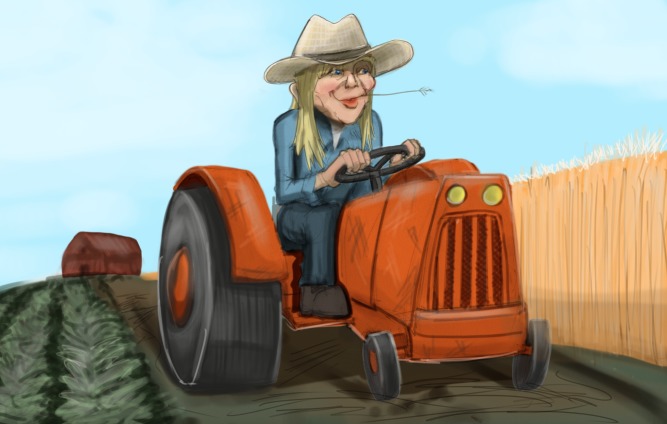
*The farmer* builds a vast storehouse of genomic data but falls short on experimental design. Prior to “planting,” researchers should define clear objectives, identify suitable analytical approaches, and consider sample-size requirements, confounding variables, and evaluation measurements. Image credit: Dan Madsen.

The farmer meticulously cultivates and gathers bushels of data (Figure 1). After a season of hard labor, she looks in her storehouse, finds enormous data files—measurements for thousands of biological features across hundreds of samples—and asks, “What now?” Unfortunately, the farmer has placed the proverbial cart before the horse: she has budgeted and planned for the seed and farmhands necessary to sow and reap crops—perhaps also developing new planting or harvesting procedures in the process—but she has failed to envision a specific use for the data. This leaves the farmer searching for ways to exploit the data; this lack of foresight may limit the return on her investment.

Over 75 years ago, experimental design practices were developed for the purpose of maximizing crop yield, usage, and distribution [2],[3]. Yet, in some cases, these lessons learned from agricultural and other research fields have not yet been adopted throughout the genomics community. Most importantly, an experimental design should be developed carefully *before* data are acquired. It should include clear objectives and/or hypotheses and delineate analytic approaches, potential confounding variables, sample-size requirements, and measures that will be used to assess the validity of the data (see Box 1) [4]. Forward planning often uncovers confounding factors or other study limitations that could minimize the value of an experiment if not anticipated and addressed in advance.

**Box 1. Essential Components of Genomics Experimental Design (*before* Acquiring the Data)**

- Specify clear objectives and/or hypotheses
- Design an experiment to directly test the specific hypotheses
- Outline analytic approaches that will be used to meet the objectives
- Anticipate potential confounding variables, sample-size requirements, and personnel needs

If possible, consult with someone who has already done the type of experiment you are attempting to do. Test the assumptions underlying your data analysis, understand the limitations of the statistical procedures you plan to use, and know what conclusions you can or cannot make from the results. For example, have study subjects been separated into sufficiently homogeneous subgroups [5]? Are there experimental/clinical/epidemiological factors that could confound your analyses? Is your study design sufficiently powered to generate a statistically significant result [6]? Will your analysis allow you to draw a decisive conclusion, or will subsequent studies be needed? And do you have access to the analytical tools and/or personnel that will be needed to interpret the data? Taking such considerations into account will help you reap greater rewards for your data-harvesting efforts.

## The Gold Miner

*“If we keep digging, eventually we will find what we are looking for.”*

**Figure 2:**
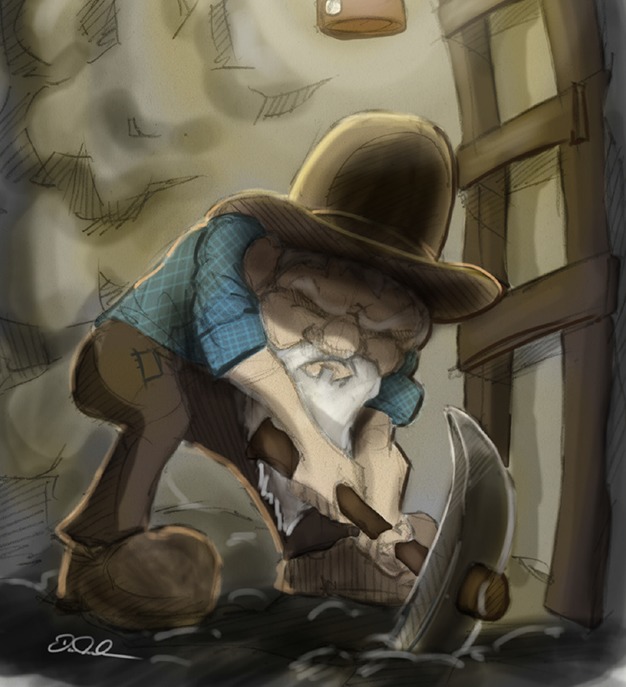
*The gold miner* keeps digging until a “significant” result surfaces. Researchers should stay true to their original experimental design, use positive and negative control experiments, and be open about the approaches that were attempted but failed. Image credit: Dan Madsen.

The gold miner relentlessly digs into the data in search of a treasure that will impress the research community (Figure 2). He hopes that with enough searching, a highly significant finding that supports his hypothesis—or any hypothesis, for that matter—will eventually surface. But even after proper planning and experimental design, results are sometimes negative. When should he give up and move to a new mine? Or, if he does find a shiny nugget, how can he be sure it's not fool's gold [7]?

Although valuable findings sometimes do arise serendipitously, it's important for genomics researchers to stay true to their original experimental design. After a dataset has been generated and the preplanned evaluations have been performed, it can be tempting to continue digging for positive results. But queries that fall outside the scope of what the original experiment was designed to address may leave the researcher standing in quicksand. By not straying from his experimental design, the gold miner can better assess when to move on from a barren mine. Additionally, because high-throughput data typically contain thousands of variables, false positive associations will inevitably arise in the data that are not valid biological findings.

Sometimes a negative result occurs simply because an experiment failed. As with laboratory research, it is important to preface critical experiments with a series of test experiments that define positive and negative controls and optimal configurations. Because the requisite statistical and bioinformatic analyses are often beyond the expertise of a basic biological scientist and because such experiments are relatively expensive, it is tempting to forego these crucial steps (see Box 2). Yet failing to run appropriate control experiments may lead to spurious associations.

**Box 2. Ensuring Sound Interpretation of Genomics Data**

- Stay true to your original experimental design
- Develop and implement negative and positive control experiments
- “Taken together, what do the data and analyses tell you?”
- Understand how statistical and computational methods should be applied
- Perform *in silico* and/or mechanistic validations

It is important to describe clearly all steps used to analyze the data—including failed attempts. For example, if you tried multiple algorithms and configuration parameters, report this in your results (if anything, as a courtesy to researchers who explore this area after you). Results that are significant only when a specific algorithm or parameters are used may be less likely to stand the test of time than those that stand up in several different analyses. Scientists should approach genomic analyses as they approach other areas of science: “taken together, what do the data and analyses tell you?”

## The Cowboy

*“We don't really understand the data, but we will go ahead and publish!”*

**Figure 3:**
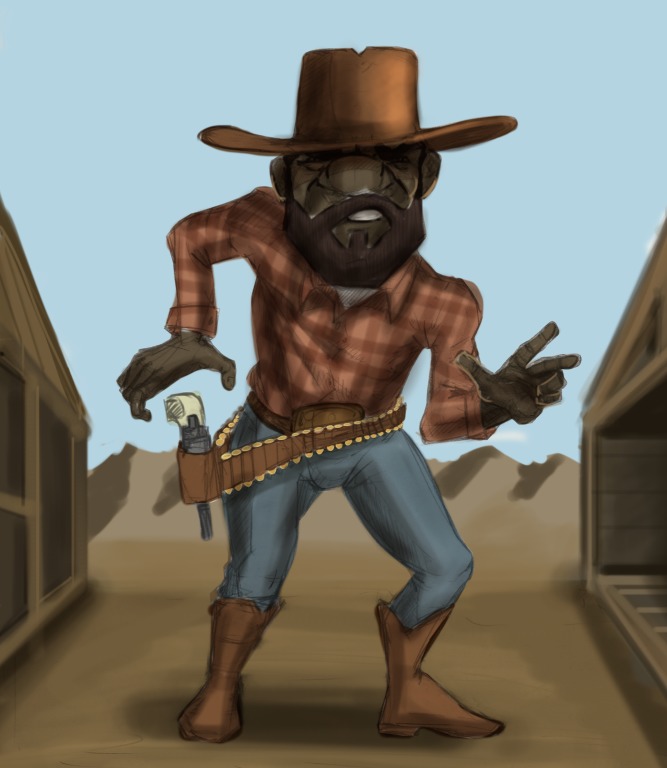
*The cowboy* wrangles data into publication without analyzing it properly. Researchers should beware of potential confounding effects and statistical biases that could lead to inappropriate conclusions. *In silico* and mechanistic validations can also overcome cowboy tendencies. Image credit: Dan Madsen.

The cowboy is always ready to push forward, shooting first and asking questions later (Figure 3). Often wrong but with no shadow of self-doubt, the cowboy places publication quantity ahead of quality. If a result appears to support his hypothesis, he wrangles it into a publication, even though he may not be sure whether the methodology was sound. If left unchecked, the cowboy's reckless behavior can mislead others on the frontier; it's only a matter of time before the Sheriff catches up and hauls him off to the jailhouse.

Before taking a publication to market, genomics researchers must exercise considerable care to ensure the validity of their results. Biologists and statisticians are often unprepared for the massive and highly complicated datasets that are generated by these new technologies. Tendencies that plague cowboys include ignoring the potential impact of “batch effects” [8],[9], focusing on only one gene or pathway of interest, applying inappropriate statistical tests, computational algorithms, or configuration parameters [10], and applying computational methods in ways that introduce bias and thus lead to overoptimistic conclusions [11]. For example, in a tumor gene-expression study, a researcher may have great success in differentiating between two apparent cancer subtypes; however, if all patients from one subtype were profiled on one day and patients from another subtype were profiled on a different day, the observed differences may be due to minor differences in sample processing rather than to an interesting biological phenomenon. Even if such biases have been avoided, it can be tempting to home in on a single gene that, for example, shows significantly different expression levels between two conditions; however, it's important to place such findings in context with other genes that may also be differentially expressed.

Finally, we stress that independent validation of all results from genomics studies is required in nearly all circumstances. This validation may be performed *in silico* using additional external datasets or simulated examples, and/or experimental validation of mechanisms inferred by the genomic findings [12]. *In silico* validation can provide a measure of confidence that a finding applies generally beyond the dataset and population from which it was derived. Mechanistic validation helps to decipher whether an observation is simply correlative or actually causal.

## The Hermit

*“I don't need help from anyone.”*

**Figure 4:**
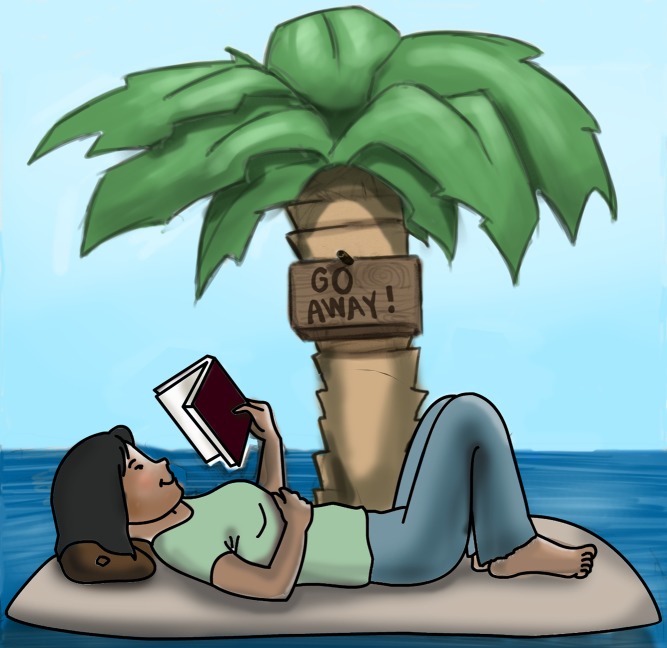
*The hermit* insists on scientific isolation and fails to realize that, in most cases, success in genomics research hinges upon collaboration among a broad range of scientists. Open-mindedness toward the conventions and idiosyncrasies of researchers from other domains is key to avoiding the hermit's existence. Image credit: Dan Madsen and Devika Joglekar.

The hermit lives in isolation, unencumbered by outsiders who might challenge her narrow view of the world (Figure 4). Blinded perhaps by distrust, over-optimism, or a false sense of superiority over those with different backgrounds or objectives, the hermit believes that her lab possesses the broad array of knowledge and skills necessary to perform any type of experiment or analysis without the need to collaborate with other scientists. The rapid pace of technological development, however, coupled with the interdisciplinary nature of genomics research, threatens to relegate the hermit to ancient history.

Effective communication and collaboration among a broad range of scientists are crucial for success in genomics [13]. Such research teams often include biologists, bioinformaticians, chemists, clinicians, computer scientists, engineers, and statisticians. Within a team, individual researchers may have very different or even contradictory objectives. For example, a statistician's typical objective is to develop new methods to address multiple embodiments of a problem; priority is placed on developing the “best” solution, not necessarily being the first to solve the problem. The biologist, by contrast, is usually intensely focused on a specific biological or medical question. Disseminating results rapidly may make the difference between publishing in a high-profile journal or one of less impact; so a “good enough” solution that can be attained quickly is often acceptable; taking time to refine and generalize methods may not be a high priority. These conflicting approaches may spawn hermitic behaviors in scientists who prefer to surround themselves only by others who share the same perspectives and objectives. Such an environment may feel safer; but valuable insight can be gained from outsiders who approach their crafts in different ways.

No individual on a team can work successfully on an island, so all researchers need to know and respect the goals, needs, and priorities of their teammates. This means, for example, that biologists may need to generate calibration data to test new technologies or validate statistical methods. Statisticians may need to develop simple solutions first that allow the biologist to move forward before the approach is generalized. Computational researchers must develop tools that are user-friendly for colleagues from different backgrounds.

When writing papers or grants, never underestimate the contributions of colleagues with different expertise; instead share primary authorship based on contribution rather than discipline, and write grants jointly that include funds for development of technology or analytical tools—not merely for their application. All researchers should be involved in projects from the earliest stages. Finally, don't jump off the wagon if technology development, data generation, or data analysis seems to be taking too long—both biological and computational experiments can encounter unexpected hurdles.

## The Master and the Servant

*“I saw this in a talk; it shouldn't be too hard for you to do the same thing.”*

**Figure 5:**
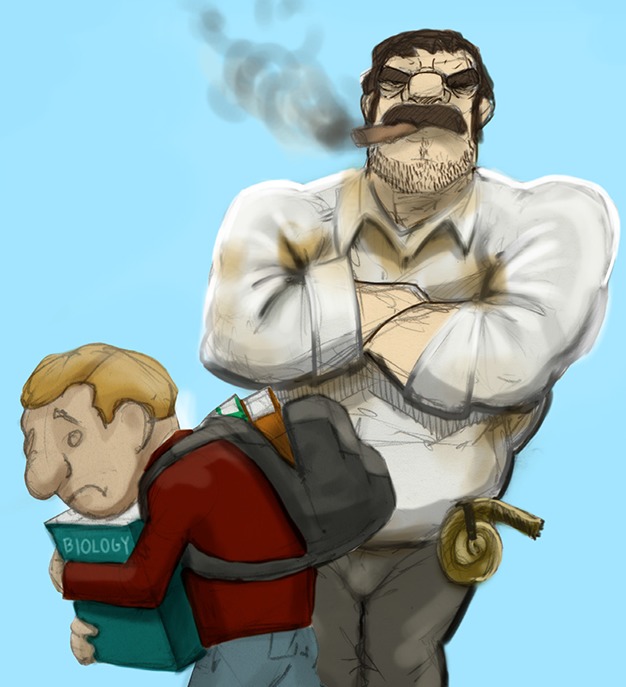
*The master* has unreasonable expectations about the expertise and time required to complete genomics research tasks; and *the servant* submits too willingly to those expectations. Front-line researchers should insist on adequate training and supervision, whereas mentors should take the long view on scientific training needs. Image credit: Dan Madsen.

When the master recognizes a need for extra hands to carry out a specific task on his ranch, he immediately recruits a new servant to address this need (Figure 5). On one hand, the master may be conversant in the general expertise of his new servant but he may fail to comprehend the effort required to produce quality results in that field. On the other hand, the servant's training might be incomplete, or he might lack independence, wanting the master to tell him exactly what to do and how to do it. It's time to educate the master and empower the servant!

Experiments or analyses often seem straightforward when described in a talk or publication, but there are always pesky underlying details. A new experimental method often does not work on the first try and may require a great deal of troubleshooting. This is also true of a computational analysis, which typically requires more than simply running a computer program with default settings. About half of computational experiments yield unexpected results [14], so even a seemingly simple analysis can take months to refine and may require input from several scientists.

Consider the example of a master with a wet laboratory background who enlists a computational servant to conduct a specific type of genomic analysis. If the master lacks expertise to advise the servant in the development and application of relevant algorithms, the servant should take the initiative to develop those skills; however, this process may take precious months of learning and troubleshooting. The master should be realistic about the time commitment required to develop new skills—and thus the potential to impact the project's timeline. Alternatively, if the servant has an inadequate understanding of the project's overall context, he may be unable to identify potential confounding factors that could affect the project's scientific validity. So master and servant should work together to ensure both have proper perspectives on the project.

Before embarking on a new genomics project, be sure to understand what experiments, analyses, and validations will be needed. Outline potential complications that could arise, formulate a realistic timeframe for project completion, and maintain open communication to address any issues that may arise. If you are mentoring a trainee who is doing something outside your expertise, make certain they are co-mentored by someone in the appropriate field. Allow the trainee time to develop the necessary background and cross-disciplinary training, even if it requires them to explore areas that are tangential to your goals or may even slow the pace of the project.

## The Jailer

*“We'll keep our data, thank you.”*

**Figure 6:**
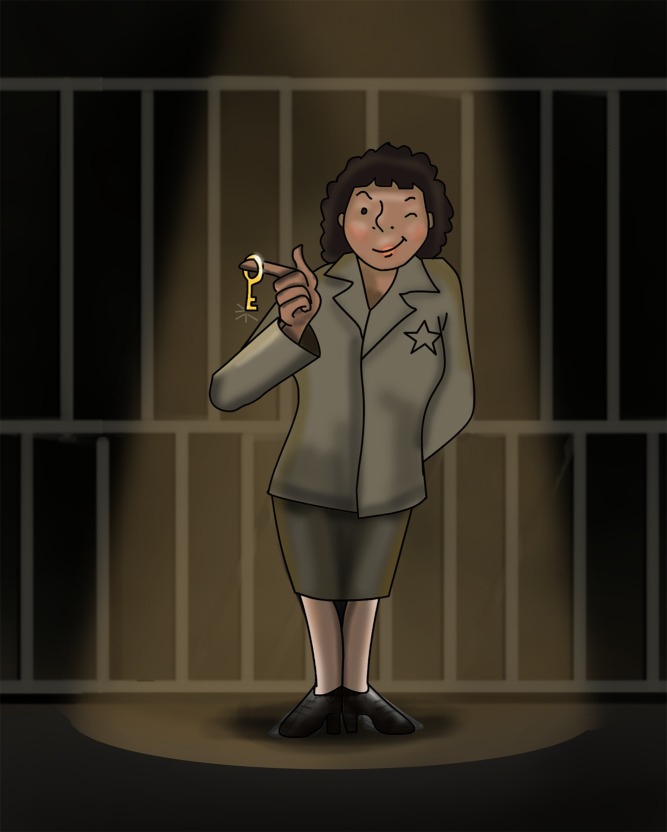
*The jailer* guards research data and tools under lock and key to maintain her competitive advantage, even though sharing would advance general scientific progress. Having published, researchers should openly share their methods and data with the community. Image credit: Dan Madsen and Devika Joglekar.

Having rustled up some data, analyzed it, and reported on it, the jailer seeks to retain full control of this precious commodity (Figure 6). She is equally as protective of computer code and scripts, which she hopes to harness for a future competitive advantage. The jailer relinquishes control of these resources only when held at gunpoint by a journal or governing body.

Freely sharing data and tools—and thus enabling your research to be reproduced and extended—are scientific and ethical responsibilities [15]. Research subjects volunteer biological specimens expecting their contributions to advance science, not necessarily the investigator's career. Taxpayers who provide funds for public research surely have similar expectations. In an attempt to advance her own status in a competitive environment, the jailer hinders scientific progress and leaves others questioning the scientific validity of her work. Don't keep the data and tools locked up!

By publishing raw data alongside a publication, researchers enable others to glean additional insights. Great value can be derived from applying new methods to existing datasets or combining data in meta-analyses [16]. Whenever possible, make all original genomics data publicly available upon publication in freely accessible databases [17],[18]. Code files should be complete, well annotated, and posted in freely accessible repositories. Data filtering, preprocessing, parameters, and analysis steps should be detailed enough that other competent researchers can reproduce the findings without the need to contact the authors. In Box 3, we describe effective practices for sharing data or code, recognizing that every study may have unique challenges that prevent data sharing in practice [19].

**Box 3. Data Sharing Practices for Genomics Researchers**

- Describe methods in sufficient detail that others can apply them
- Make raw and processed data available in public repositories like Gene Expression Omnibus, Database of Genotypes and Phenotypes, or Sequence Read Archive
- Share code and execution scripts in version-control repositories like GitHub or SourceForge
- Annotate code or script files

## Danger Warning

We urge you to be on the lookout for these character traits that are far too common on the genomics frontier. Be aware of your own personal tendencies toward these potentially damaging behaviors. Many stem from a lack of understanding or relevant training (such as “the farmer,” “the hermit,” “the cowboy,” and “the master”), whereas some arise from a lack of awareness of customs or standards (specifically, “the gold miner,” “the servant,” and “the jailer”).

Success in genomics requires a competent and unified team with a broad range of skills and talent working to a well defined “battle plan.” Communal success should take priority over individual notoriety. We look forward to many hoedowns where we'll celebrate each other's achievements in taming this exciting frontier!
